# ASSOCIATIONS BETWEEN PULMONARY FUNCTION AND PHYSICAL REHABILITATION OUTCOMES AMONG OLDER ADULTS

**DOI:** 10.1093/geroni/igae098.3805

**Published:** 2024-12-31

**Authors:** Lindsey Mathis, Na Sun, Simon Ho, Lane White, Odessa Addison, Douglas Savin, Jason Falvey

**Affiliations:** University of Maryland Baltimore, Baltimore, Maryland, United States; University of Maryland Baltimore, Baltimore, Maryland, United States; University of Maryland Baltimore, Baltimore, Maryland, United States; University of Maryland Baltimore, Baltimore, Maryland, United States; University of Maryland School of Medicine, Baltimore, Maryland, United States; University of Maryland Baltimore, Baltimore, Maryland, United States; University of Maryland School of Medicine, Baltimore, Maryland, United States

## Abstract

Physical rehabilitation is crucial for older adults with functional impairments, as persistent impairments are linked to increased risks of hospitalization, mobility disability, and mortality. Impaired pulmonary function and associated symptoms of dyspnea may limit participation in rehabilitation programs, but it is unclear whether these impairments negatively impact outcomes. To answer this question, our analysis included 819 community-dwelling rehabilitation users (63% female; weighted n=3,882,191) from Round 5 (2015) of the National Health and Aging Trends Study (NHATS). Peak expiratory flow (PEF) categorized participants into “normal,” “moderately impaired,” or “severely impaired” lung function. Participants reported whether they met rehabilitation goals during their latest episode of care (yes/no). Multivariable survey-weighted logistic regression, adjusting for demographics and medical complexity, was used to estimate these relationships. In our sample, 71% had normal PEF, 22% had moderate PEF impairment, and 7% had severe PEF impairment. Severe impairment had 57% lower odds (aOR=0.4, 95% CI 0.2-0.9) and moderate impairment had 35% lower odds (aOR=0.6, 95% CI 0.4-1.0) of meeting rehabilitation goals compared to normal PEF. Similar trends were observed when limiting rehab users to only musculoskeletal condition (e.g., joint replacement). This study found that nearly 4 million older adults in the U.S. use rehabilitation annually, with 29% having impaired lung function. Our data suggest impairments in PEF may limit progress with rehabilitation, even for those with musculoskeletal indications, and thus suggests care pathways to more optimally manage older adults with limited lung function in rehabilitation settings may be needed to help them achieve their goals.

